# Seroepidemiology of infection with *Toxoplasma gondii *in healthy blood donors of Durango, Mexico

**DOI:** 10.1186/1471-2334-7-75

**Published:** 2007-07-13

**Authors:** Cosme Alvarado-Esquivel, Miguel Francisco Mercado-Suarez, Alfredo Rodríguez-Briones, Laura Fallad-Torres, Julio Octavio Ayala-Ayala, Luis Jorge Nevarez-Piedra, Ehecatl Duran-Morales, Sergio Estrada-Martínez, Oliver Liesenfeld, José Ángel Márquez-Conde, Sergio Arturo Martínez-García

**Affiliations:** 1 Faculty of Medicine, Juárez University of Durango State (UJED). Durango, Mexico; 2 Mexican Institute of Social Insurance (IMSS), Durango, Mexico; 3 State Center for Blood Transfusion, Secretary of Health. Durango, Mexico; 4 Institute for Scientific Research, UJED. Durango, Mexico; 5 Institute for Microbiology and Hygiene, Campus Benjamin Franklin, Charité Medical School Berlin, Germany

## Abstract

**Background:**

*Toxoplasma gondii *(*T. gondii*) infection in blood donors could represent a risk for transmission in blood recipients. There is scarce information about the epidemiology of *T. gondii *infection in blood donors in Mexico. Therefore, we sought to determine the prevalence of *T. gondii *infection and associated socio-demographic and behavioral characteristics in a population of healthy blood donors of Durango City, Mexico.

**Methods:**

Four hundred and thirty two blood donors in two public blood banks of Durango City, Mexico were examined for *T. gondii *infection between August to September 2006. Blood donors were tested for anti-*T. gondii *IgG and IgM antibodies by using enzyme-linked immunoassays (Diagnostic Automation Inc., Calabasas, CA, USA). Socio-demographic and behavioral characteristics from each participant were also obtained.

**Results:**

Thirty two (7.4%) of 432 blood donors had IgG anti-*T. gondii *antibodies. Eight (1.9%) of them had also IgM anti-*T. gondii *antibodies. Multivariate analysis using logic regression showed that *T. gondii *infection was associated with the presence of cats at home (adjusted OR = 3.81; 95% CI: 1.45–10.01). The age group of 45–60 years showed a significantly higher frequency of *T. gondii *infection than the group of 25–34 years (p = 0.02). Blood donors without education had a significantly higher frequency of infection (15.8%) than those with 13–19 years of education (4.5%) (p = 0.04). Other characteristics of blood donors including male gender, consumption of undercooked meat or blood transfusion did not show an association with infection.

**Conclusion:**

The prevalence of *T. gondii *infection in healthy blood donors of Durango City, Mexico is lower than those reported in blood donors of south and central Mexico, and is one of the lowest reported in blood donors worldwide. *T. gondii *infection in our blood donors was most likely acquired by contact with cats. Prevalence of infection increased with age and decreased with educational level.

## Background

Estimates indicate that up to one third of the world's population is infected by *T. gondii *[1,2]. Most infections in immunocompetent humans are asymptomatic and in up to 10% of infected individuals cervical lymphadenopathy or ocular disease occur [2]. Primary infection acquired during pregnancy could result in congenital toxoplasmosis [2,3]. In immunocompromised individuals, *T. gondii *infections could cause central nervous system disease as encephalitis or brain abscess [2,4]. Routes of parasite transmission in humans include 1) ingesting food or water that is contaminated with oocysts shed by cats; 2) eating undercooked or raw meat containing tissue cysts [2,5,6] and; 3) transplantation and blood transfusion [7-10].

The epidemiology of *T. gondii *infection in general, and in blood donors in particular has been poorly studied in Mexico. There is not any surveillance study or *T. gondii *screening program in blood donation, women in child bearing age or immunosuppressed patients. Studying a blood donor population is a valuable approach to determine epidemiological characteristics in adults of a community and might provide findings that could be found in the general adult population of the same community. There is a lack of information about the epidemiology of *T. gondii *infection in blood donors in northern Mexico. Therefore, we performed a cross-sectional study to determine the prevalence of *T. gondii *infection in blood donors of Durango City, Mexico and to identify characteristics of blood donors associated with seropositivity.

## Methods

### Study design and study population

We performed a cross sectional study (observational, prospective and descriptive survey) in the two largest blood banks of Durango City, Mexico. These blood banks were: blood bank 1, the General Hospital Blood Bank of the Mexican Institute of Social Insurance, and blood bank 2, the State Center for Blood Transfusion of the Secretary of Health. Inclusion criteria for the study subjects were: 1) voluntary blood donors; 2) aged 18 years and older; and 3) who accepted to participate in the study. All samples were routinely tested for antibodies against human immunodeficiency virus (HIV), hepatitis C virus (HCV), and *Treponema pallidum*, and hepatitis B virus surface antigen (HBsAg) in parallel to testing for antibodies against *T. gondii*. None of the blood donors were seropositive for HIV, HCV, HBsAg and *Treponema pallidum*. Two hundred and one blood donors of the first blood bank and 231 donors of the second blood bank attended from August to September 2006 were enrolled consecutively. In total 432 voluntary healthy blood donors participated in the study. Blood banks in Durango City are public, attend mostly low income blood donors, do not pay any blood donation, and give donated blood or blood products in a free manner to hospitals.

### Ethical aspects

This study was approved by the Institutional Ethical Committee. The purpose and procedures of the study were explained to all participants, and a written informed consent was obtained from all of them.

### Socio-demographic and behavioral data

We used a standardized questionnaire to explore the characteristics of the blood donors. Socio-demographic data including age, birth place, residence place, marital status, occupation, educational level, and housing conditions index were obtained from all participants. Housing conditions index was obtained by using the Bronfman's criteria [11]. Briefly, five variables were evaluated: number of persons in the house, number of rooms in the house, material of the floor of the house, availability of drinkable water, and form of elimination of excreta. Contributing and confounding risk factors of behavioral data including blood transfusion or transplant history; animal contacts, cleaned up cat excrement, foreign travel, kind of meat consumption (pork, lamb, beef, goat, boar, chicken, turkey, rabbit, deer, squirrel, horse, fish and iguana), raw or undercooked meat consumption, unpasteurized milk or milk products consumption, untreated water consumption, dried or cured meat eaten (chorizo, ham, sausages or salami), unwashed raw vegetables or fruits consumption, contact with soil (gardening or agriculture), and eating outside of the home (at least once a year) from all blood donors were obtained.

### Laboratory tests

Serum samples were obtained by centrifugation of fresh whole blood of the donors. The serum samples were transported from the blood banks to the Laboratory of Microbiology of the Faculty of Medicine (Juarez University of Durango State) where the samples were frozen down and kept stored at -20°C until analyzed. Serum samples of blood donors were analyzed for anti-*T. gondii *IgG antibodies by a commercially available enzyme immunoassay "*Toxoplasma *IgG" kit (Diagnostic Automation Inc., Calabasas, CA, USA). In addition, sera positive for anti-*T. gondii *IgG antibodies were further analyzed for anti-*T. gondii *IgM antibodies by a commercially available enzyme immunoassay "*Toxoplasma *IgM" kit (Diagnostic Automation Inc., Calabasas, CA, USA). Both tests were performed in the Laboratory of Microbiology of the Faculty of Medicine following the instructions of the manufacturer. For both IgG test and IgM test, we calculated the Toxo G index and Toxo M index, respectively. These indexes are calculated for each determination by dividing the mean values of each sample by the cut-off calibrator mean value. A sample was considered positive for IgG or IgM when a Toxo G index or a Toxo M index was equal or greater than 1.0 (>8 IU/ml). A positive IgG test with a negative IgM test in a donor was interpreted as a chronic infection. A positive IgM test with a positive IgG test in a donor was interpreted as probability of recent infection.

### Statistical analysis

The statistical analysis was performed with the aid of the software Epi Info version 3.3.2 and SPSS version 7.5 (SPSS Inc. Chicago, IL. USA). For calculation of the sample size we used a reference seroprevalence of 8.9% [12] as expected frequency of the factor under study, 10,000 as the size of population from which the sample was selected, a worst acceptable result of 12%, and a confidence level of 95%. The result of the calculation was 314 subjects. Descriptive statistics were used for numerical (mean and median) and categorical (frequency or percentage) variables. We used the Mantel-Haenszel test, and the Fisher exact test (when cells values were less than 5) for comparison of the frequencies among groups. For ordinal variables we used the *X*^2 ^for trend. Bivariate and multivariate analyses were used to assess the association between the characteristics of the subjects and *T. gondii *seropositivity. As a criterion for inclusion of variables in the multivariate analysis, we considered variables with a p value equal or less than 0.2 obtained in the bivariate analysis to allow potential confounding. Adjusted odd ratio (OR) and 95% confidence interval (CI) were calculated by multivariate analysis using multiple, unconditional logistic regression. A p value less than 0.05 was considered statistically significant.

## Results

### Seroprevalence of anti-*T. gondii *antibodies

Seroprevalence of anti-*T. gondii *IgG antibodies in blood banks 1 and 2 were 9 % (18/201) and 6.1% (14/231), respectively. No statistically significant difference was found among the prevalences of anti-*T. gondii *IgG antibodies in each blood bank (p = 0.25). Anti-*T. gondii *IgM antibodies were found in 2 of the 18 IgG positive donors from blood bank 1, and in 6 of the 14 IgG positive donors from blood bank 2. Overall, thirty two (7.4%) of 432 blood donors were positive for anti-*T. gondii *IgG antibodies and eight (1.9%) of them were also positive for anti-*T. gondii *IgM antibodies.

### Socio-demographic characteristics and other possible risk factors associated with seropositivity

General socio-demographic characteristics of the 432 blood donors studied are shown in Table 1. The mean age of the blood donors was 30.4 +/- 8.8 years (median: 29 years; range: 18 to 60 years). The majority of blood donors were born in Mexico and resided in urban areas of Durango State. Most blood donors were males, have studied up to 12 years, and used to live in regular or good housing conditions. The highest frequency of infection (11%) was found in the age group of 35–60 years while the lowest frequency (4.3%) in the age group of 25–34 years. The difference among these frequencies (11% vs 4.3%) was statistically significant (p = 0.02). There was a trend of higher seroprevalence of anti-*T. gondii *IgG antibodies as educational level decreased (p = 0.04). Blood donors without education had a significantly higher frequency of infection (15.8%) than those with 13–19 years of education (4.5%) (p = 0.04).

**Table 1:** Socio-demographic characteristics of the 432 blood donors in Durango, Mexico.

	Donors studied^a^		Donors positive for anti-*T. gondii *antibodies	
Characteristic	No.	%	No.	%
Gender				
Male	374	86.6	28	7.5
Female	58	13.4	4	6.9
Age groups (years)				
18–24	135	31.3	10	7.4
25–34	161	37.3	7	4.3
35–60	136	31.4	15	11
Born in Mexico				
Yes	427	99.8	31	7.3
No	1	0.2	0	0
Residence Place				
Durango State	424	98.1	31	7.3
Other Mexican State	7	1.6	0	0
Abroad	1	0.2	0	0
Residence area				
Urban	304	71.5	20	6.6
Suburban	45	10.6	6	13.3
Rural	76	17.9	5	6.6
Educational level				
No education	38	9	6	15.8^e^
Up to 12 years (College)	295	69.7	21	7.1
13–19 years (Undergraduate)	88	20.8	4	4.5
Graduate	2	0.5	0	0
Occupation				
No laborer^b^	207	48.3	14	6.8
Laborer^c^	143	33.3	12	8.4
Other/unemployed^d^	79	18.4	5	6.3
Housing conditions index				
Good	215	62.3	16	7.4
Regular	95	27.5	10	10.5
Bad	35	10.1	0	0

^a^Blood donors with available data.

^b^Non laborer = Professional, businessman, employee.

^c^Laborer = Farmer and worker in factory or construction areas.

^d^Other/unemployed = student and housewife.

^e^Statistically significant difference among the educational level groups by *X*^2 ^for trend: p = 0.04.

In the bivariate analysis, six variables were identified as possible risk factors associated with *T. gondii *infection: 1) cats at home (p = 0.01); 2) cats in the neighborhood (p = 0.19); and 3) living in a house with soil floors (p = 0.19). In addition, we found two variables that showed possible negative association with *T. gondii*: 1) pork meat consumption (p = 0.05); 2) turkey meat consumption (p = 0.01); and 3) sausage consumption (p = 0.2). Other sociodemographic and behavioral characteristics of the blood donors did not show any possible association with *T. gondii *infection. Table 2 shows the results of the bivariate analysis of selected variables and the results of *T. gondii *seropositivity. Further analysis by using logistic regression revealed that only the variable cats at home was significantly associated with *T. gondii *seropositivity (adjusted OR = 3.81; 95% CI: 1.45–10.01) (Table 3).

**Table 2:** Bivariate analysis of putative risk factors for infection with *T. gondii *in the 432 blood donors from Durango, Mexico.

Characteristic	Blood donors^a^No. (%)	Positive test for anti-*T gondii *antibodies No. (%)	P value
Pork meat consumption			
Yes	361 (84.9)	21 (5.8)	0.05
No	64 (15.1)	8 (12.5)	
Turkey meat consumption			
Yes	126 (29.5)	3 (2.4)	0.01
No	301 (70.5)	29 (9.6)	
Sausage consumption			
Yes	367 (85.7)	25 (6.8)	0.2
No	61 (14.3)	7 (11.5)	
Degree of meat cooking			
Raw or undercooked	10 (2.3)	1 (10.0)	0.53
Well done	422 (97.7)	31 (7.3)	
Unwashed raw vegetable or fruit consumption			
Yes	59 (13.8)	3 (5.1)	0.59
No	367 (86.2)	29 (7.9)	
Cats at home			
Yes	116 (27)	15 (12.9)	0.01
No	314 (73)	17 (5.4)	
Cleaning cat feces			
Yes	46 (11.1)	4 (8.7)	0.46
No	367 (88.9)	27 (7.4)	
Cats in the neighborhood			
Yes	155 (36.2)	15 (9.7)	0.19
No	273 (63.8)	17 (6.2)	
Gardening or agriculture			
Yes	123 (29.4)	7 (5.7)	0.45
No	296 (70.6)	23 (7.8)	
Blood transfusion			
Yes	6 (1.4)	0 (0)	0.49
No	419 (98.6)	30 (7.2)	
Soil floors			
Yes	11 (2.8)	2 (18.2)	0.19
No	389 (97.3)	28 (7.2)	

^a^Blood donors with available data.

**Table 3:** Multivariate analysis of selected characteristics of the 432 blood donors and their association with *T. gondii *infection.

Characteristic^a^	Adjusted odds ratio^b^	95% Confidence Interval	P value
Pork meat consumption	0.38	0.11–1.28	0.12
Turkey meat consumption	0.34	0.09–1.23	0.1
Cats at home	3.81	1.45–10.01	0.006
Cats in the neighborhood	0.64	0.25–1.67	0.36
House with soil floors	2.94	0.26–32.8	0.38
Sausage consumption	0.7	0.22–2.26	0.55

^a^The variables included were those with a p ≤ 0.20 obtained in the bivariate analysis.

^b^Adjusted by age, housing conditions index, and the rest of characteristics included in this table.

## Discussion

In the present study, we found a 7.4% seroprevalence of *T. gondii *infection in blood donors of Durango, Mexico. This prevalence could be considered low and questions on the sensitivity of the test might rise. However, we think that the sensitivity of the tests was very good and the results were reliable. The sensitivity of the IgG test is 94%. Quality control of both IgG and IgM test runs showed valid results. The low prevalence of seropositivity for *T. gondii *found is comparable with that detected in other healthy population of Durango City using a different detection method [13]. This prevalence is much lower than that reported in the southern Mexican State of Yucatan [14], and the central Mexican State of Jalisco [15] where researchers found that 69% and 29% of blood donors were positive for anti-*T. gondii *antibodies, respectively. Our prevalence found in Durango City is also much lower than those reported in blood donors from other Latin American countries, as Brazil [16], Cuba [17], and Chile [18]. Similarly, our prevalence is much lower than that found in blood donors in Malaysia [19], Saudi Arabia [20], Czech Republic [21], and Mali [22] were prevalences varied from 21% to 52.1%. In contrast, our prevalence was comparable with the 9% prevalence found in blood donors of Loei Province, Thailand [23]. It is possible that differences in the characteristics of the blood donors and differences in the environments might contribute to explain the lower prevalence of *T. gondii *infection found in our blood donor population than those reported in blood donors from southern and central Mexico or other countries. Most of our blood donors belonged to a low socio-economic level and eating meat is not a frequent practice among them. In addition, consumption of undercooked or raw meat is rarely found among our blood donors. Environmental characteristics of Durango City as a dry climate, and a high altitude may also contribute to explain the low frequency of infection. This explanation is supported by previous observations that prevalence of *T. gondii *infection in populations living in dry climates was lower than those living in other climates [2,24,25], and lower in populations living in high altitudes than those in low altitudes either in humans [26,27] or in animals [28].

With respect to the demographic characteristics of the blood donors, we observed that the frequency of seropositivity increased with age, and this observation agrees with those reported in other studies [16,20]. In addition, we observed that seropositivity to *T. gondii *decreased as educational level in donors increased (p = 0.04). To the best of our knowledge, a similar observation in blood donors had not been reported. This finding deserves further study in which additional results might confirm or challenge our finding. We can not state that water influenced our result since untreated water consumption or other water variables were not associated with seropositivity in our study. Socio-economic status does not explain our finding since most donors belonged to a low income population. We speculate that high education is linked to good hygienic sanitary practices thereby reducing the transmission of the parasite. Male and female blood donors showed comparable prevalences of *T. gondii *infection, and this result does not support previous observations that showed a higher prevalence of infection in male than female blood donors [16,23]. The number of female donors in this study was much lower than that of male donors. Therefore, further studies are needed to elucidate risk factors associated with seropositivity in female donors. The knowledge of factors associated with seroconversion in women is particularly important during their reproductive age in order to design preventive measures and avoid acute infections during pregnancy. The fact that in this study the variable "cats at home" was associated with infection indicates that *T. gondii *infection in our infected population might have occurred by ingesting parasite oocysts in contaminated food or water. Contact with cats has not always been associated with *T. gondii *seropositivity in epidemiology studies, as shown in a previous study in blood donors and HIV patients [19] or in pregnant women [13]. In contrast, in our study we did not observe an association of seropositivity with consumption of any meat explored indicating that meat consumption was not relevant in parasite transmission in the blood donors studied. Meat consumption has been found to be an important factor in parasite transmission in several studies [13,29]. However, in some studies no association between *T. gondii *infection and meat consumption has been found [12,19]. The very low number of donors with anti-*T. gondii *antibodies found in this study reduces the statistical power to find further associations between seropositivity and the epidemiological characteristics in blood donors. Certainly by increasing the sample size the statistical power increases and some risk factors with borderline significance might turn out to become significant. This is especially interesting for risk factors including meat consumption, [13,29], and soil floors [13].

Interestingly, nearly 2% of our blood donors had also IgM antibodies against *T. gondii*. The absence of this infection marker in subjects with anti-*T. gondii *IgG antibodies indicates a chronic infection but its presence does not necessarily indicate an acute infection. We performed detection of IgM antibodies only in IgG positive samples because the presence of IgM antibodies alone is rarely seen. Anti-*T. gondii *IgG antibodies appears very early after infection [30], therefore, the window period between the appearance of IgM and the appearance of IgG is extremely short and the probability to find an IgM positive/IgG negative infected subject seems to be quite low to financially justify a systematic IgM screening in a cross sectional study. In addition, seropositivity to IgM alone is not considered an acceptable diagnostic criterion for acute infection. Anti-*T. gondii*-specific IgM antibodies are detectable early after infection and can persist for prolonged times after infection [2,31]. IgM-positive donors with parasitemia may hold a potential for parasite transmission by blood transfusion. We were unable to judge whether a fraction of our blood donors might represent a risk group for parasite transmission by blood transfusion as reported previously [8,15].

## Conclusion

We concluded that the prevalence of *T. gondii *infection in blood donors of Durango City, Mexico is low as compared with those reported in central and south Mexico and the majority of other countries. *T. gondii *infection in our blood donors was most likely acquired by contact with cats. Infection increased with age and decreased with educational level.

## Competing interests

The author(s) declare that they have no competing interests.

## Authors' contributions

CAE conceived and designed the study protocol, participated in the coordination and management of the study, performed the laboratory tests and data analysis, and wrote the manuscript. MFMS, ARB, JOAA, LJNP, EDM, and JAMC applied the questionnaires and performed the data analysis. SEM performed the statistical analysis. LFT performed the laboratory tests. OL designed the study protocol, performed the data analysis, and wrote the manuscript. SAMG performed the data analysis.

## Pre-publication history

The pre-publication history for this paper can be accessed here:


